# miRVaS: a tool to predict the impact of genetic variants on miRNAs

**DOI:** 10.1093/nar/gkv921

**Published:** 2015-09-17

**Authors:** Sophia Cammaerts, Mojca Strazisar, Jenne Dierckx, Jurgen Del Favero, Peter De Rijk

**Affiliations:** 1 Applied Molecular Genomics Unit, Department of Molecular Genetics, VIB, Antwerp, 2610, Belgium; 2 University of Antwerp, Antwerp, 2610, Belgium; 3 Multiplicom N.V., Niel, 2845, Belgium

## Abstract

Genetic variants in or near miRNA genes can have profound effects on miRNA expression and targeting. As user-friendly software for the impact prediction of miRNA variants on a large scale is still lacking, we created a tool called miRVaS. miRVaS automates this prediction by annotating the location of the variant relative to functional regions within the miRNA hairpin (seed, mature, loop, hairpin arm, flanks) and by annotating all predicted structural changes within the miRNA due to the variant. In addition, the tool defines the most important region that is predicted to have structural changes and calculates a conservation score that is indicative of the reliability of the structure prediction. The output is presented in a tab-separated file, which enables fast screening, and in an html file, which allows visual comparison between wild-type and variant structures. All separate images are provided for downstream use. Finally, we tested two different approaches on a small test set of published functionally validated genetic variants for their capacity to predict the impact of variants on miRNA expression.

## INTRODUCTION

MicroRNAs (miRNAs) are short non-protein-coding RNAs that regulate expression of their target genes mainly by translational repression or mRNA destabilization (1). Targeting of metazoan miRNAs is mostly defined by incomplete complementarity of the mature miRNA sequence to transcripts; the seed region, nt 2–7 of the mature miRNA, is considered to be a major determinant for target recognition (2). As a consequence miRNAs can target a large set of mRNAs (3) and mRNAs can be regulated by several miRNAs, leading to a complex regulation network (4).

Genes encoding miRNAs can be located individually or in clusters and can be intergenic or can be located within host genes. In the canonical biogenesis pathway, the primary transcript (pri-miRNA) is cleaved sequentially to release the mature miRNA(s). In the first processing step, which takes place in the nucleus, the Microprocessor, consisting of a complex between the ribonuclease Drosha and its cofactor DGCR8, cleaves the pri-miRNA to free the precursor miRNA (pre-miRNA) (5–7). The cleavage site of the Microprocessor is determined by ssRNA–dsRNA junctions at both ends of the miRNA hairpin embedded within the pri-miRNA and the distances between the two junctions. The complex cleaves at a position that is located approximately 11 nt from the junction between the lower stem and the flanking regions and approximately 22 nt from the junction between the upper stem and the terminal loop of the hairpin (8–10). In addition to the importance of the hairpin structure within the primary transcript, sequence motifs were also shown to play a role in Drosha processing (11). The pre-miRNA is transported to the cytosol by Exportin 5 (12). In the second maturation step, Dicer cleaves off the terminal loop after recognizing both ends of the pre-miRNA, resulting in a mature miRNA duplex (13–15). After the duplex is loaded into an Argonaute protein, one of the two strands is discarded. The remaining mature miRNA strand guides Argonaute to its target mRNAs (16).

Many proteins regulate the maturation process of miRNAs by inhibiting or enhancing the action of Drosha or Dicer or by affecting the stability of miRNAs (17). At both processing steps the hairpin can be cleaved at multiple positions, thereby creating alternative mature miRNAs, called isomiRs, differing in length and sequence. IsomiRs can also be generated by addition or removal of nucleotides of the 3′ end of the mature miRNA, by editing of the mature miRNA sequence or due to the presence of genetic variants (18). An overview of miRNA biogenesis and its regulation can be found in the review by Ha and Kim (19).

Genetic variants in or near miRNA genes can affect expression and function of the nearby miRNA gene. Variants in the mature miRNA sequence can result in altered target specificity, as was demonstrated by a variant in the seed sequence of *MIR627* (20). Mutations in miRNA genes have been shown to cause human disease, such as mutations in the seed sequences (5p and 3p) of *MIR96* causing non-syndromic hearing loss (21,22). These mutations lower the expression of the mature miRNA. For one mutation it was shown that this is due to a change in the secondary structure of the hairpin, as a double mutant with restored predicted secondary structure also had restored expression levels (22). A similar structure dependent change in expression was found for a variant in the mature sequence of *MIR125* (23). Variants located outside the mature miRNA or hairpin sequence can also result in altered expression of the mature miRNA, as was shown for different variants just outside the precursor sequence of *MIR137* (24,25) and for a variant located 199 nt downstream of the *MIR124-1* hairpin sequence (26).

Several approaches exist to predict the secondary structure of an RNA molecule. The most commonly used method is the prediction of the optimal structure based on minimal free energy (MFE) (27). This method presumes that there is a single optimal conformation and that the methods and thermodynamic parameters used are without error. Other potential or competing structures can be taken into account by computing a partition function, which sums the contribution of all structures at equilibrium, weighted by their probabilities (28). The maximal expected accuracy (MEA) structure is the structure that maximizes the expected base-pair accuracy based on this partition function (29). The ensemble of possible secondary structures can also be represented by the centroid structure prediction: the structure that has the smallest total base-pair distance to the sampled structures of that ensemble (30,31).

When assessing the potential impact of a variant on the miRNA secondary structure for a large number of variants, such as those generated using massively parallel sequencing, using the currently available tools requires a lot of manual work. Online versions of mfold and RNAfold (32,33), commonly used tools for this type of predictions, require individual RNA sequences as input for each wild-type and variant miRNA. Results then need to be visually compared and interpreted by the researcher for each variant separately. The miRNASNP database website offers a tool to predict secondary structures of miRNAs with variants (34). However this also requires submission of wild-type and variant sequences and visual comparison and cannot be used for batch searches. To automate these time-consuming and error-prone manual processes, we developed miRVaS, a tool that, starting from a list of variants, determines the location of the variant relative to functional regions within the miRNA hairpin and that predicts the impact of the variant on the secondary structure of the miRNA.

## MATERIALS AND METHODS

### RNA structure prediction

RNAfold (35) (version 2.1.5) is used by miRVaS to predict the secondary structure of RNA sequences. It is run with parameters to calculate the MFE structure, the partition function and MEA and centroid structures (-p and –MEA), adding dangling energies for the bases adjacent to a helix on both sides (-d2). No postscript figure is generated (–noPS).

### miRNA database file generation

miRNA database files for the test set were generated by extracting genomic coordinates, hairpin and mature miRNA sequences from miRBase (36) v20 and the human genome file (hg19). Loop regions were extracted from the miRBase v20 miRNA.str file. In cases where the mature miRNA sequence overlaps with the predicted loop region, the loop annotation was shifted outside the mature region for the miRNA database file.

### Visualization

RNA secondary structures are visualized using VARNA (37) (version 3.9) using the naview algorithm (-algorithm naview). Output is generated in png and svg format. The svg files are post-processed by miRVaS to allow automatic scaling to the page size.

### Illustration of miRVaS using test set

In order to test the usefulness of miRVaS annotation and to test parameters that may predict an effect on miRNA expression due to genetic variants, we collected a list of miRNA variants of which the effect on the expression of the nearby miRNA has been investigated in cell lines. The studies used had to fulfil following criteria for inclusion: (i) genetic variant located within or near miRNA gene (< 0.5 kb), (ii) variant and wild-type miRNA constructs expressed in a cell line (only one variant per construct), (iii) expression of the relevant miRNA in the cell line was assessed by qPCR or northern blot and (iv) genomic location of the variant and alternative allele could be derived from the text. In cases where the location of the variant was given relative to miRNA precursors, without a reference to the miRBase version used or sequences in the text, we searched the sequence of the oldest version of miRBase containing the miRNA gene to deduct the genomic coordinates of the variant. Genomic coordinates of variants were extracted from 26 papers. The resulting variant list (in zero based half open coordinates format) was used as an input for miRVaS, together with the human genome file (hg19) and the miRNA database file based on miRBase v20. Predictions were run with up- and downstream flanks of 100 nt surrounding the hairpin. Calls were calculated based on reported results (categorized as changed expression or no change in expression) and based on miRVaS output (ΔG or highest structural impact region).

For the ΔΔG (defined as ΔG of variant structure – ΔG of wild-type structure) approach, we tested whether a variant that alters the stability of the predicted (MFE) structure affects expression of the miRNA *in vivo*. Following calls were made for the ‘|ΔΔG| > 0 strategy’: true positive (TP): |ΔΔG| > 0 and the variant induces the expression of the miRNA to change *in vivo*; true negative (TN): |ΔΔG| = 0 and the variant does not induce the expression of the miRNA to change *in vivo*; false positive (FP): |ΔΔG| > 0 and no expression change *in vivo*; false negative (FN): |ΔΔG| = 0 and expression changes *in vivo*. The ‘density interval’ was determined by plotting the density of the ΔΔG for variants inducing expression change *in vivo* and variants inducing no expression change (using ggplot2 (38)) and determining the intersection points of the curves. Calls were made as follows: TP: ΔΔG < −2.103226 or ΔΔG > 4.324731 (intersection points of density curves) and the variant induces an expression change *in vivo*; TN: ΔΔG ≥ −2.103226 and ΔΔG ≤ 4.324731 and no expression change *in vivo*; FP: ΔΔG < −2.103226 or ΔΔG > 4.324731 and no expression change *in vivo*; FN: ΔΔG ≥ −2.103226 and ΔΔG ≤ 4.324731 and expression changes *in vivo*.

For the hairpin approach, we tested whether variants causing a predicted structural change within the hairpin (i.e. the pre-miRNA plus a flanking stem region) affect the expression of the miRNA. Following calls were made for each structure representation (centroid, MEA, MFE): TP: structural change with the highest impact is located within the hairpin and the expression of the miRNA changes *in vivo*; TN: structural change with the highest impact is confined to a region outside the hairpin and no expression change *in vivo*; FP: structural change with the highest impact is located within the hairpin and no expression change *in vivo*; FN: structural change with the highest impact is confined to a region outside the hairpin and expression changes *in vivo*.

Because predictions were run with flanks of 100 nt, variants located further than 100 nt from the hairpin were treated as negatives, i.e. for the ΔΔG approach with the ‘|ΔΔG| > 0 strategy’: |ΔΔG| = 0, ‘density interval strategy’: ΔΔG ≥ −2.103226 and ΔΔG ≤ 4.324731 or for the hairpin approach: structural change with the highest impact is located outside the hairpin. Sensitivity was defined as TP/(TP+FN)*100; specificity as TN/(TN+FP)*100. For statistical analysis a two-tailed Fisher's exact test was performed.

## RESULTS

miRVaS predicts the impact of genetic variants on miRNAs. It annotates the location of the variant relative to functional regions within the miRNA hairpin (seed, mature, loop, hairpin arm, flanks) based on an accompanying database of miRNA genes with all functional regions. Database files with human miRNAs based on miRBase (36) are included in the download from the miRVaS website. These databases, e.g. for other species, can also be generated by a database conversion tool implemented within miRVaS. Next, the impact of the variant on the secondary structure of the miRNA is predicted by extraction of the sequence of the miRNA hairpin and flanking regions from the genome sequence, introduction of the variant in the sequence and subsequent prediction of the structure of both wild-type and variant sequences using RNAfold (35). The resulting string form structures are compared, after realignment in case of insertions or deletions. The location of all bases that changed secondary structure (paired versus unpaired) is annotated relative to the functional regions within the miRNA. Comparison and annotation is done for centroid, MEA and MFE structure representations.

In addition to impact prediction, miRVaS also creates html files with drawings of the predicted structures so that the changes can be visually compared. It uses VARNA (37) to create secondary structure drawings highlighting: (i) the genetic variant, (ii) important regions within the miRNA (hairpin, mature miRNA, seed, terminal loop) and (iii) the nucleotides with structural changes between the wild-type and variant miRNA. miRVaS can thus be used for batch predictions of the impact of genetic variants, generating tabular output, html files and separate image files. The tool can be installed locally and can be operated using a graphical user interface or via command line. Alternatively, users can access an online version of miRVaS for impact prediction of human miRNA gene variants (http://mirvas.bioinf.be/analyse.html).

### Input

miRVaS requires three input files: (i) a variant file in tab-delimited format with the genomic coordinates (zero based half open format), reference and alternative allele of the variants or in VCF format; (ii) a genome file and (iii) a miRNA database file describing all functional regions within the miRNAs. A detailed description of these files is provided in the miRVaS manual. The human genome file (build hg19, build hg38) and a miRNA database file based on miRBase (v20, v21) (36) are distributed with the software. miRVaS includes a database conversion utility to convert gff3 files, as provided by miRBase, into the correct format. This can be used to add newer versions of miRBase, miRNA data of other species or novel miRNA data from other sources.

For most miRNAs the exact length of the primary transcript is unknown and hence setting the correct flank sizes surrounding the miRNA hairpin is not possible. Accordingly, the user can choose the flank size used for the predictions by setting the parameters Upstream flank (5p) and Downstream flank (3p). Default miRVaS settings are up- and downstream flanks of 100 nt, based on the suggestion that to establish overexpression of mature miRNAs *in vivo*, approximately 100 nt of flanking regions surrounding the hairpin should be included in the miRNA construct (39).

### Output

**Figure 1.**
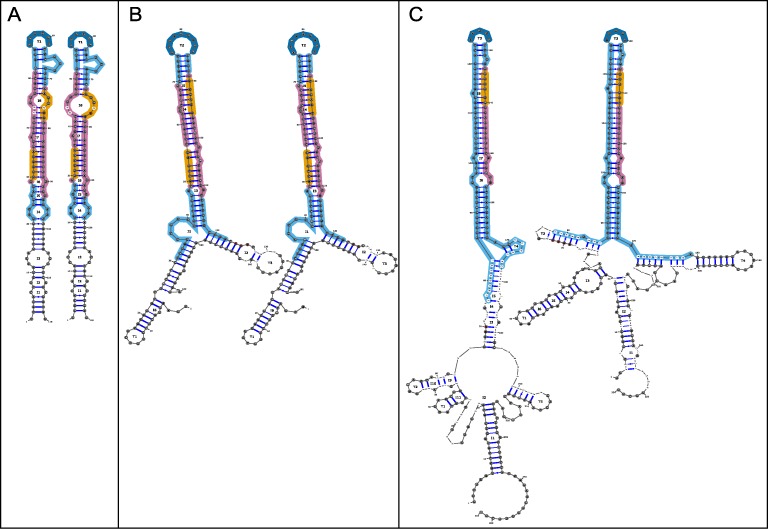
Visual output of miRVaS. The left figure in each panel represents the wild-type miRNA, the right figure represents the same miRNA with the variant. Orange regions: seed sequence, magenta: mature miRNA, dark blue: terminal loop, cyan: hairpin. The variant is highlighted in red in the wild-type and the variant miRNA, bases with structural changes are highlighted in black. (**A**) Structure prediction for a SNP within the seed sequence. Up- and downstream flanks used for the prediction: 20 nt/20 nt. The SNP enlarges an interior loop. (**B**) Structure prediction for a deletion of one base within the flanking region. Up- and downstream flanks used for the prediction: 30nt/40nt. The deletion does not change the secondary structure drastically. (**C**) Structure prediction for an insertion of two bases in the flanking region. Up- and downstream flanks used for the prediction: 75 nt/75 nt. This insertion causes structural changes in the lower hairpin arms and in the flanking regions.

miRVaS creates output for all miRNA genes in the miRNA database file that are located in the vicinity of the specified variants, separated in different rows for the same variant. As such, the user does not need to know upfront whether the tested variants are located near a miRNA gene (or which one), which can be useful when working with large variant lists. Firstly, a tab-separated file is created containing variant location annotation relative to functional regions within the miRNA, annotation of structural changes for centroid, MEA and MFE predictions, delta free energies (ΔG) of reference and variant structures and the frequency of the MFE structure in the thermodynamic ensemble for all variants specified in the input file. Additional features are described below. Secondly, an html file is made containing links to all structure predictions with, for each variant, the centroid, MEA and MFE predictions for reference and variant next to each other, allowing easy visual comparison. Functional regions are highlighted in different colours: in orange: seed region; in magenta: mature miRNA; in dark blue: terminal loop; in cyan: hairpin. The variant is indicated in red, structural changes are indicated by black bases. For insertions and deletions, the bases surrounding the inserted or deleted region are also in red in the images to clearly indicate the position of change (Figure 1). Thirdly, a directory containing separate html files per variant and image files for all predictions in png and svg format is generated.

### Annotation

**Figure 2.**
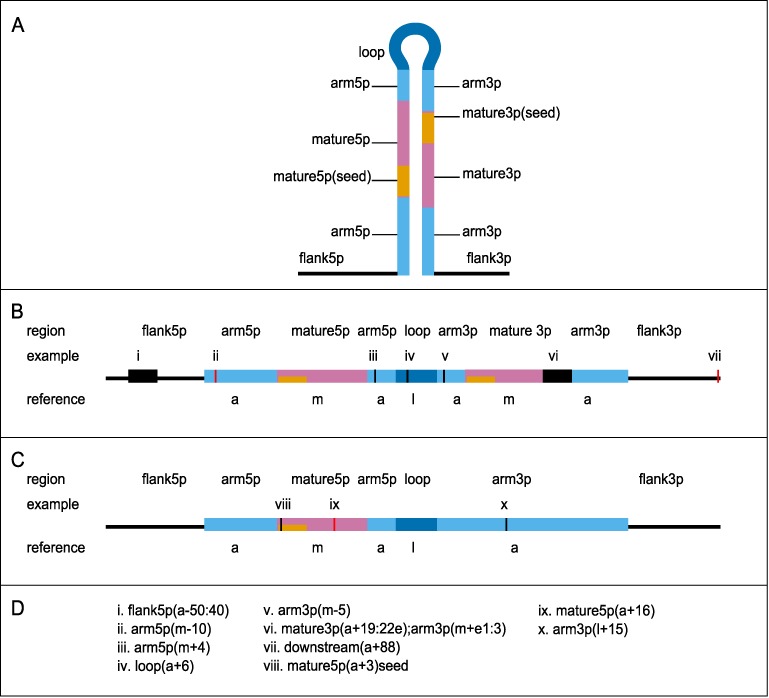
Annotation scheme for variant location and structural changes. Annotations are made in the format: region (reference ± start:stop). A detailed description of the annotation can be found in the Results section. (**A**) Schematic overview of the miRNA hairpin including flanks. Functional regions used for annotation are highlighted according to the colour scheme used by miRVaS. The length of the flanking region is determined by the user. (**B**) A schematic view of structural and sequence changes in a miRNA gene with two mature sequences (5p and 3p). Black bars depict regions with structural change, sequence changes are highlighted in red. Regions are denoted in the upper part of the figure and correspond to regions in Panel A. Reference regions are shown below the hairpin (a: arm, m: mature, l: loop). (**C**) A schematic view of structural and sequence changes in a miRNA gene with one mature sequence (5p). (**D**) Annotation of examples of sequence and structural changes that are depicted in Panels B and C: (i) a structural change in the 5p flanking region covering bases 50–40 upstream of the hairpin arm; (ii) a sequence change in the lower 5p hairpin arm, 10 bases upstream of the 5p mature, (iii) a structural change in the upper 5p hairpin arm, 4 bases downstream of the mature 5p; (iv) a structural change in the loop region, 6 bases downstream of the upper 5p arm; (v) a structural change in the upper 3p hairpin arm, 5 bases upstream of the mature 3p; (vi) a structural change spanning the end of the mature 3p (bases 19–22 downstream of the upper 3p arm, base 22 is the last base of the 3p mature sequence) and the beginning of the lower 3p arm (1st–3rd base downstream of the mature 3p); (vii) a sequence change outside the given flank region, 88 bases downstream of the lower 3p hairpin arm; (viii) a structural change within the seed sequence, 3rd base downstream of the lower 5p hairpin arm; (ix) a sequence change within the 5p mature sequence, 16 bases downstream of the lower 5p hairpin arm; (x) a structural change within the 3p hairpin arm, 15 bases downstream of the calculated loop region.

Location and structure annotations in the output are provided in the following format: region (reference ± start:stop), which is illustrated in Figure 2. The first part of the annotation—the region—indicates in which functional segment of the miRNA gene the sequence change and/or structural change is located. The part between parentheses indicates the position of the change within this functional segment: it states the position (start, stop) starting from the border with the flanking region indicated by reference.

The different regions are: (i) upstream/downstream: outside the given flank sizes for the tested miRNA, but less than 2000 nt outside any miRNA hairpin (in this case, location annotation is given, but no structure predictions can be done); (ii) flank: within the given flanking region; (iii) arm: within the hairpin; (iv) loop: within the terminal loop; (v) mature: within the mature miRNA sequence. For changes within nt 2–7 of the mature sequence, the mature region annotation is appended with ‘seed’.

The reference regions are: a: hairpin arm, m: mature region, l: loop. When a change reaches or crosses the end or the start of a region, an ‘e’ is appended to the base position. All annotations are relative to the orientation of the gene.

### Additional features

Next to the ΔG energies and frequencies of the structures calculated by RNAfold, and the provided location annotation, structural impact annotation and visualization, we devised two other parameters that may be of use to researchers who want to prioritize variants with a potential functional impact.

*Conservation*. As the miRNA structure is of paramount importance in the biogenesis of the mature miRNA, it is unlikely that the hairpin structure would be completely absent or largely changed *in vivo*. When the predicted secondary structure of the hairpin changes by using different flank sizes, this could reflect the unreliability of the secondary structure prediction. To assess the extent of conservation of the secondary structure of the miRNA hairpin, miRVaS calculates whether the hairpin secondary structure for a prediction with specific flank sizes is the same as the prediction of the hairpin structure without any flank. When the hairpin is not conserved, the prediction is marked as ‘changed’, along with the number of changed bases within the hairpin.

#### Highest structural impact prediction

miRVaS annotates and highlights structural changes that are predicted to occur in the miRNA when the variant is introduced in the sequence. To enable the user to quickly assess for a list of variants where the impact for each of the miRNAs is located, miRVaS displays the ‘most important’ region that is structurally changed due to the presence of the variant, with importance defined from most to least important: seed > mature > arm > loop > flank. This information is given in tabular as well as the html output for centroid, MEA and MFE predictions in the ‘highest impact’ fields.

An example of miRVaS tabular output for a variant in *MIR125* and a description of the different fields can be found in Table 1.

**Table 1.** miRVaS tabular output: description of the different fields

chromosome	chr19	Genomic location of the variant in zero based half open coordinates, type of variant, reference and alternative allele. Type can be snp (SNP), ins (insertion) or del (deletion).
begin	52196527	
end	52196528	
type	snp	
ref	G	
alt	T	
mir_location	mature5p(a+8)	Location of the variant relative to the functional regions within the tested miRNA.
		Example: the variant is located in the 5p mature sequence, 8 nt downstream of the lower 5p hairpin arm.
mir_name	hsa-mir-125a	Tested miRNA.
MFE_highest_impact*	seed	Highest region in which a structural change is present for the MFE structure. Order: seed > mature > arm > loop > flank.
		Example: the highest region with structural changes is the seed region.
MFE_impact*	mature5p(a+2,5,8:9)seed&mature3p(a+14:15,19:20)	Describes all structural changes in the variant structure compared to the reference structure for the MFE structure.
		Example: structural changes are within the 5p mature and seed sequence (positions 2, 5 and 8–9, counting from the lower 5p hairpin arm) and within the 3p mature sequence (positions 14–15 and 19–20, counting from the upper 3p hairpin arm).
ref_mfefreq	0.000285697	Frequency of the MFE structure for the reference allele.
var_mfefreq	0.0000634247	Frequency of the MFE structure for the variant allele.
MFE_conservation*	conserved	Whether the hairpin structure is conserved with given flank sizes compared to when using no flanks. When not conserved, the number of bases with changed structure is given.
		Example: the structure of the hairpin is conserved.
ref_MFE_deltaG*	−91.2	Free energy of the MFE structure prediction for the reference allele (kcal/mol).
var_MFE_deltaG*	−85.5	Free energy of the MFE structure prediction for the variant allele (kcal/mol).

As an example, the output for a variant within *MIR125* is described (for up- and downstream flanks of 100 nt). Rows and columns are transposed for clarity. *Columns are also calculated for centroid and MEA predictions (not shown).

### Illustration of miRVaS using functionally studied test cases

As shown in Figure 1 and Table 1, miRVaS provides a wealth of information that can be used to assess the potential functional impact of miRNA gene variants. It can be difficult to assess what kind of structural change may likely result in an expression change *in vivo*. For this reason we tested two different approaches to predict the impact of variants using a set of functionally investigated variants as a reference. To obtain this test set, we searched the literature for human genetic variants in or near miRNA genes for which the effect on the expression of the nearby miRNA was functionally investigated. For 41 genetic variants the impact of the variant on the expression of the 32 nearby miRNA(s) was studied in cell lines (21–23,25,26,40–60). The set of validated miRNA gene variants to date is still very limited, and is in line with the range of variants that was reported by Gong *et al*. (61). Finding the correct flank sizes introduces a problem, as for most miRNA genes the exact primary transcript lengths are unknown. Due to the fact that several validated variants are located outside the pre-miRNA and that it has been suggested to include flanks of approximately 100 nt surrounding the hairpin when establishing overexpression of miRNAs (39), we ran miRVaS with flank sizes of 100 nt.

The first approach we tested to predict variant-induced miRNA expression changes, the ‘ΔΔG approach’, is based on the hypothesis proposed by Gong *et al*. (61). The original hypothesis poses that variants inducing destabilization of the hairpin reduce the expression of the mature miRNA *in vivo*, while variants that increase the stability increase the expression of the mature miRNA. Because we are interested in whether a variant may affect expression of the miRNA or not, regardless of the direction, we tested whether a variant that affects the stability of the predicted structure (in any direction, |ΔΔG| > 0) affects the expression of the miRNA *in vivo*. However, this approach does not seem to be a good predictive model, as the ΔΔG and the absence or presence of expression changes are not associated (*P* = 0.3825, Table 2, Supplementary Table S1).

**Table 2.** Performance of miRVaS determined by the test set for the ΔΔG approach with two different ΔΔG strategies (|ΔΔG| > 0 or density interval)

	|ΔΔG| > 0	Density interval
TP	25	11
FP	11	0
TN	3	14
FN	3	17
Sensitivity (%)	89	39
Specificity (%)	21	100
*P*-value	0.3825	0.0075

*P*-value: two-tailed *P*-value of Fisher's exact test.

The ‘|ΔΔG| > 0 strategy’ may be too strict as small deviations in ΔG may not have an effect on expression, but a large test set is not available for optimization of this parameter. For this reason we plotted the densities of the ΔΔG for variants that induce a miRNA expression change *in vivo* and variants that have no effect on miRNA expression (Supplementary Figure S1). Though the density plots largely overlap, the plot for the variants that affect miRNA expression is more spread. Therefore, we used the intersection points of the curves as thresholds (‘density interval strategy’). This strategy performed better (*P* = 0.0075, Table 2, Supplementary Table S1). Although the specificity is now much higher (100%), this is at the cost of a very low sensitivity (39%).

As an alternative, we devised an approach that takes into account the location of the predicted structural change, i.e. the ‘hairpin approach’, and tested whether it may be useful in the impact prediction of variants. In this approach, we assessed whether a variant that induces a predicted structural impact change within the hairpin (calculated by miRVaS as the ‘highest structural impact’ parameter) results in expression changes *in vivo*. The miRNA hairpins deposited in miRBase contain the pre-miRNA and a small part of the flanking region (on average a hairpin stem of 10 nt outside the pre-miRNA). This point, the ssRNA–dsRNA junction approximately 11 nt upstream of the pre-miRNA, together with the ssRNA–dsRNA junction at the terminal loop region, is recognized by the Microprocessor to be able cleave the pre-miRNA at the correct position (8–10). As the structure of the hairpin is paramount for its correct processing, structural changes within the hairpin are likely to influence the expression of the miRNA by affecting Drosha or Dicer processing. Structural changes that are only located outside this region are less likely to affect Microprocessor processing, though it is a possibility, for example for structural changes just outside the hairpin that affect pri-miRNA recognition. To explore and compare the effects of using alternative structure prediction methods, we tested this approach on the results of the centroid, MEA and MFE structure predictions. Despite being a simplified model, the hairpin approach turns out to be a good predictive model in our limited test set, especially when using the MFE structure prediction (*P* = 0.0008, Table 3, Supplementary Table S2). The sensitivity and specificity for the MFE structure are 71% and 86%, respectively. However, due to the limited number of variants that are currently functionally validated, we do not consider this result definitive, until larger variant sets become available to train and validate the software on. Consequently, this approach is not included in the miRVaS software as a direct prediction. Nevertheless, we do support the use of this approach, as it simplifies variant prioritization and performs well on the currently available test set.

**Table 3.** Performance of miRVaS determined by the test set for the hairpin approach

	CEN	MEA	MFE
TP	19	19	20
FP	2	2	2
TN	12	12	12
FN	9	9	8
Sensitivity (%)	68	68	71
Specificity (%)	86	86	86
*P*-value	0.0025	0.0025	0.0008

CEN: centroid, MEA: maximal expected accuracy, MFE: minimal free energy.

## DISCUSSION

Several online databases gather information on variants in or near miRNAs, such as miRvar (62), miRNA SNiPer (63), PolymiRTS (64) and miRNASNP (34). The miRNASNP database is an extensive resource that includes annotation of variant location and presents secondary structure predictions for wild-type and variant miRNAs. It also presents a call about the potential impact of the variant on the expression of the miRNA based on the difference in free energy between the wild-type and variant structures. However, it only does this for variants within the pre-miRNA and the database cannot be used for batch searches, making investigations of multiple variants laborious. For novel variants, databases cannot be used to extract variant information. Commonly used online available tools for secondary structure impact prediction of miRNA variants are mfold and RNAfold (32,33). However, these tools are not designed to assess the effects of variants on (mi)RNAs and as such require the user to prepare wild-type and variant sequences manually and run the program separately for each. The resulting structures then need to be compared and interpreted manually for each miRNA. The miRNASNP website includes, next to tools to assess the impact of miRNA seed variants and 3′ UTR variants on miRNA targeting, a tool for secondary structure impact prediction for wild-type and variant (precursor) miRNAs, but here again the user needs to prepare and submit individual sequences and compare the results (34). The RNAsnp web server is focused on prediction of RNAs with variants as well (65). Users can supply genomic coordinates of the query sequence and of one or more variants of interest within that sequence. However, insertions and deletions cannot be tested and the user still needs to run the analysis for each miRNA separately and compare the results. Thus, using these tools in the context of miRNA gene variant impact prediction is labor-intensive and error-prone, especially when more than one variant needs to be investigated, for example when exploring the numerous variants obtained with massively parallel sequencing platforms. Furthermore, the majority of these tools are not designed specifically for miRNA research and as such it is up to the user to determine where functionally important regions are located within the structure prediction. Though a tool for automation of prediction of the impact of batches of (novel and known) variants in the 3′ UTR of target genes on miRNA binding does exist (mrSNP (66)), such a user-convenient tool is still lacking for the secondary structure prediction of miRNAs with variants. To meet these needs, we developed miRVaS.

miRVaS greatly facilitates and speeds up the evaluation of the impact of genetic variants on miRNAs. The tool annotates the location of the variant relative to functional regions within the miRNA gene, describes all structural changes between the structure predictions of the miRNA with and without the variant and generates visual output, while eliminating the need to prepare FASTA files. The tool can run large numbers of genetic variants simultaneously, thereby significantly reducing the manual workload for the user. Furthermore, the user does not need to know which miRNAs are located in the vicinity of the variant, as the tool explores the potential impact on any nearby miRNA gene(s). Therefore, the tool is of great use to assess and prioritize variant lists originating from genome sequencing or RNA sequencing experiments, as no prior filtering or analysis needs to be done to assess which genes may be affected by the variants. Of note, the use of miRVaS is not limited to variants within the human genome or to miRNA genes deposited in miRBase, but can be expanded to miRNAs in other species and to novel miRNA genes, depending on the research focus.

To date only a limited number of published studies have described the effect of a genetic variant on the expression of the nearby miRNA gene and so the set of available variants used for testing miRVaS was small. We believe it would be beneficial to the community if information about functionally validated miRNA gene variants would be included in existing miRNA gene variant databases. Bias towards the publication of positive results (i.e. variants that affect miRNA expression) in the test set can be expected. Nevertheless, we did find negative results (variants with no effect on the expression of the nearby miRNA gene), including a study where none of the tested variants had an effect on expression (42) and four studies reporting a variant with no effect on expression (22,44,52,60), but most studies only reported positive results. In addition, negative reported results are more uncertain as they may result from differences too small to be picked up by the technologies used (e.g. northern blot), or because under the conditions studied, no effects could be seen, but that effects are visible under other conditions. This complicated picture is illustrated well by rs2910164 located in *MIR146A*: Jazdzewski *et al*. found a difference in expression of miR-146a due to the variant in U2OS cells, Shen *et al*. also found a difference in expression, but in the opposite direction in MCF-7 cells, while a study by Lung *et al*. did not find changed expression of miR-146a in 293FT cells (43,45,55).

Based on the currently available set of genetic variants for which the impact on the miRNA expression was tested in cell lines, we illustrated the use of miRVaS. Two different approaches were tested to interpret the results. The first approach, based on the difference in free energy in the secondary structure prediction induced by the variant, did not perform well. The second approach was based on the hypothesis that structural changes within the hairpin may result in *in vivo* expression changes. Though this is a simplified approach, and though it is very likely that the impact of variants inducing large structural changes just outside the hairpin region will be missed, when applied to our test cases, this assumption did perform well. Of the three different structure representations, the MFE structure scored best. As such, this result is a validation of the usefulness of the MFE structure as the most commonly used structure representation in the context of miRNA variant impact prediction. Due to the lack of an extensive test set to train the software and the lack of full understanding of the biogenesis of miRNAs, these results cannot be considered definitive. Therefore the ‘hairpin approach’ is not incorporated into the tool as a direct prediction, although we do believe it is a good approach to prioritize variants based on the results of the test set. To allow investigation of alternative assumptions or a multistage approach, miRVaS presents all gathered data, such as location of the variant, all structural changes and a highest impact parameter, but the final decision of the relevance of the variants is up to the researcher.

Several assumptions could be used to determine which variants are most likely to have a functional impact on miRNAs. A first possible focus is to look at the variant location annotation, as variants within the mature or seed sequence, whether or not they are predicted to change the structure of the hairpin, might change the targeting of the miRNA. Next, the researcher can use the highest impact classifications to determine whether the impact is located within or outside the hairpin and assume that a structural change within the hairpin is more likely to have an effect on expression than a change confined to a region outside the hairpin. Since the structure representations are predicted, it is important that the researcher can assess the reliability of the structure predictions. This can be done by taking into account the calculated conservation parameter, for instance by filtering out predictions with a lot of changes within the hairpin when using a specific flank size, or by looking for consistent changes when running miRVaS with different flank sizes or between different structure representations.

Functional validation of the effect of variants on miRNAs is expensive and time-consuming. With the increasing, massively parallel sequencing driven, identification of genetic variants near and in miRNA genes, it is crucial to predict upfront their functional effect, enabling selection of the most interesting variants for further detailed functional studies.

## AVAILABILITY

miRVaS can be downloaded from http://mirvas.bioinf.be/ or can be used directly on the website. miRNA database files (based on miRBase v20, v21), human genome files (hg19, hg38), the variant file for the test set (‘testset_input.tsv’) and the manual can also be accessed from this website.

## Supplementary Material

SUPPLEMENTARY DATA
