# Supplementary material for: miRVaS: a tool to predict the impact of genetic variants on miRNAs
Source: Nucleic Acids Res. 2015 Sep 17;44(3):e23. doi: 10.1093/nar/gkv921 (PMC4756848; doi:10.1093/nar/gkv921)
Supplement: SUPPLEMENTARY DATA [file supp_44_3_e23__index.html]

miRVaS: a tool to predict the impact of genetic variants on miRNAs — miRVaS: a tool to predict the impact of genetic variants on miRNAs — SUPPLEMENTARY DATA 

# miRVaS: a tool to predict the impact of genetic variants on miRNAs

## SUPPLEMENTARY DATA

- SUPPLEMENTARY DATA
