# Supplementary material for: miRVaS: a tool to predict the impact of genetic variants on miRNAs
Source: Nucleic Acids Res. 2015 Sep 17;44(3):e23. doi: 10.1093/nar/gkv921 (PMC4756848; doi:10.1093/nar/gkv921)
Supplement: SUPPLEMENTARY DATA [file supp_gkv921_nar-02234-met-n-2015-File004.pdf]

Supplementary Data

Supplementary Figure 1. Density plots for ΔΔG of MFE predictions of variants with and without an effect on miRNA expression. Intersection points between the curves are shown by the vertical lines.

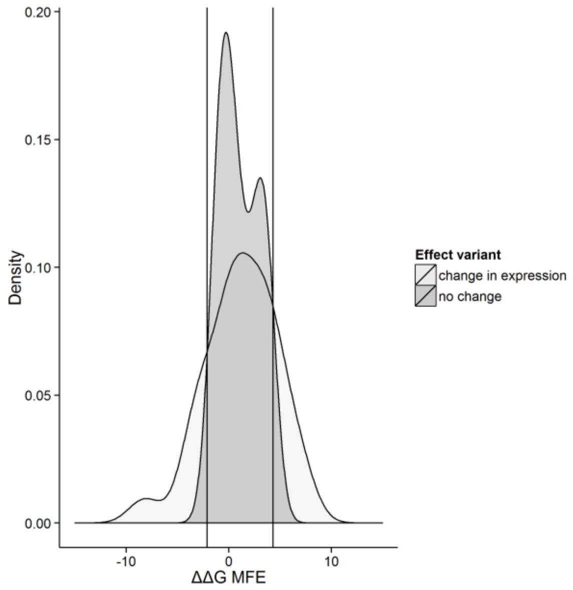

Supplementary Table 1. Results of the analysis of the test set with the ΔΔG approach for two different ΔΔG thresholds. PMID: Pubmed ID of the study validating the effect of the variant on expression of the miRNA. Effect: extracted from the referenced study. Location: miRVaS location annotation of the variant relative to the miRNA indicated in the column 'miRNA'. ΔΔG(MFE): ΔG of variant MFE structure – ΔG of wild-type MFE structure. Call A: based on the ΔΔG approach with the |ΔΔG| > 0 strategy, Call B: based on the ΔΔG approach with the density interval strategy. Variant chr13:50623101-50623102 G>A was tested twice since its effect was validated on two different miRNAs. For variant chr5:159912417-159912418 C>G, two studies found a difference in expression (a third did not), the effect was categorized as 'changed'.

Chr	Begin	End	Ref	Alt	PMID	Effect	miRNA	Location	ΔΔG(MFE)	Call A	Call B
chr1	9211781	9211782	C	T	25242229	change in expr	hsa-mir-34a	arm3p(m-e9)	0.60	TP	FN
chr1	98511730	98511731	C	T	24888363	change in expr	hsa-mir-137	flank5p(a-4)	1.20	TP	FN
chr1	98511733	98511733		CCGCTGCCGCTGCTA	24888363	change in expr	hsa-mir-137	flank5p(a-7:6)	-8.20	TP	TP
chr2	241395502	241395503	T	C	23272122	change in expr	hsa-mir-149	arm3p(m+10)	-2.20	TP	TP
chr3	49058127	49058128	C	A	20167074	change in expr	hsa-mir-191	arm5p(m-1e)	4.60	TP	TP
chr5	148808389	148808390	G	A	16778182	no change	hsa-mir-143	flank5p(a-91)	3.90	FP	TN
chr5	148810075	148810076	C	A	16778182	no change	hsa-mir-145	upstream(a-133)	0.00	TN	TN
chr5	148810203	148810204	G	A	16778182	no change	hsa-mir-145	flank5p(a-5)	1.10	FP	TN
chr5	159912417	159912418	C	G	18474871;18660546; 22711332	change in expr	hsa-mir-146a	mature3p(a+4)seed	-3.00	TP	TP
chr5	168195355	168195356	G	A	23566829	change in expr	hsa-mir-218-2	flank5p(a-96)	1.80	TP	FN
chr6	52009266	52009267	C	T	18356149	no change	hsa-mir-206	flank3p(a+35)	-0.40	FP	TN
chr7	129414552	129414553	A	G	22038834	change in expr	hsa-mir-96	mature3p(a+6)seed	3.90	TP	FN
chr7	129414573	129414574	A	G	22038834	no change	hsa-mir-96	loop(a+e1)	-1.00	FP	TN
chr7	129414595	129414596	G	T	19363479	change in expr	hsa-mir-96	mature5p(a+6)seed	6.70	TP	TP
chr7	129414596	129414597	C	T	19363479;22038834	change in expr	hsa-mir-96	mature5p(a+5)seed	7.20	TP	TP
chr8	9760698	9760699	G	C	22430032	change in expr	hsa-mir-124-1	downstream(a+199)	0.00	FN	FN
chr9	73424963	73424964	G	A	26056285	no change	hsa-mir-204	mature5p(a+5)seed	2.70	FP	TN
chr9	96938219	96938220	C	A	16778182	no change	hsa-let-7a-1	flank5p(a-19)	3.70	FP	TN
chr9	139565149	139565150	A	G	20621067	change in expr	hsa-mir-126	flank3p(a+12)	-3.10	TP	TP
chr10	53059405	53059406	T	C	25683625	change in expr	hsa-mir-605	arm3p(m+2)	-2.60	TP	TP
chr10	102734777	102734778	C	G	24743625	change in expr	hsa-mir-608	mature5p(a+22)	3.00	TP	FN
chr10	135061111	135061112	C	T	23334589	change in expr	hsa-mir-202	arm5p(m-15)	0.70	TP	FN
chr12	62997345	62997346	G	C	16778182	no change	hsa-let-7i	upstream(a-120)	0.00	TN	TN
chr12	81329535	81329536	A	C	24503492	change in expr	hsa-mir-618	arm3p(l+25)	-3.80	TP	TP
chr13	50623101	50623102	G	A	16251535	change in expr	hsa-mir-16-1	flank3p(a+7)	0.00	FN	FN
chr13	50623101	50623102	G	A	16251535	change in expr	hsa-mir-15a	downstream(a+153)	0.00	FN	FN
chr16	69967004	69967005	C	A	20358594;22012839	change in expr	hsa-mir-140	arm5p(m-1e)	2.50	TP	FN
chr17	57918726	57918727	A	G	16778182	no change	hsa-mir-21	flank3p(a+29)	-1.30	FP	TN
chr19	52196135	52196136	G	A	18356149	change in expr	hsa-let-7e	flank3p(a+19)	-0.70	TP	FN
chr19	52196408	52196409	G	A	21788734	change in expr	hsa-mir-125a	flank5p(a-98)	1.40	TP	FN
chr19	52196452	52196453	T	C	21788734	no change	hsa-mir-125a	flank5p(a-54)	-1.00	FP	TN
chr19	52196527	52196528	G	T	17400653	change in expr	hsa-mir-125a	mature5p(a+8)	5.70	TP	TP
chr21	17912066	17912067	C	G	16778182	no change	hsa-let-7c	flank5p(a-81)	2.70	FP	TN
chr22	46509539	46509540	C		16778182	no change	hsa-let-7b	flank5p(a-26)	1.30	FP	TN
chr22	46509676	46509677	G	C	16778182	no change	hsa-let-7b	flank3p(a+29)	3.40	FP	TN
chr22	46509701	46509702	G	A	16778182	no change	hsa-let-7b	flank3p(a+54)	0.00	TN	TN
chrX	49779217	49779218	C	G	19568434;19617315	change in expr	hsa-mir-502	arm5p(m-3)	3.80	TP	FN
chrX	135633050	135633051	T	G	19617315	change in expr	hsa-mir-934	mature5p(a+e1)	2.10	TP	FN

chrX	145075803	145075804	C	G		19617315	change in expr	hsa-mir-890	arm3p(l+23)	3.50	TP	FN
chrX	145078732	145078733	A	G		19617315	change in expr	hsa-mir-892b	mature3p(a+15)	4.70	TP	TP
chrX	146353878	146353879	A	G		19568434;19617315	change in expr	hsa-mir-510	mature3p(a+2)seed	3.90	TP	FN
chrX	146353920	146353921	C	T		19617315	change in expr	hsa-mir-510	arm5p(m-4)	-0.60	TP	FN

Supplementary Table 2. Results of the analysis of the test set with the hairpin approach. HI columns: highest structural impact as calculated by miRVaS. Call: based on the hairpin approach.

Chr	Begin	End	Ref	Alt	PMID	Effect	miRNA	Location	HI CEN	HI MEA	HI MFE	Call CEN	Call MEA	Call MFE
chr1	9211781	9211782	C	T	25242229	change in expr	hsa-mir-34a	arm3p(m-e9)	arm	arm	arm	TP	TP	TP
chr1	98511730	98511731	C	T	24888363	change in expr	hsa-mir-137	flank5p(a-4)	flank	flank	arm	FN	FN	TP
chr1	98511733	98511733		CCGCTGCCGCTGCTA	24888363	change in expr	hsa-mir-137	flank5p(a-7:6)	flank	flank	arm	FN	FN	TP
chr2	241395502	241395503	T	C	23272122	change in expr	hsa-mir-149	arm3p(m+10)	nochange	nochange	nochange	FN	FN	FN
chr3	49058127	49058128	C	A	20167074	change in expr	hsa-mir-191	arm5p(m-1e)	mature	mature	mature	TP	TP	TP
chr5	148808389	148808390	G	A	16778182	no change	hsa-mir-143	flank5p(a-91)	flank	flank	flank	TN	TN	TN
chr5	148810075	148810076	C	A	16778182	no change	hsa-mir-145	upstream(a-133)				TN	TN	TN
chr5	148810203	148810204	G	A	16778182	no change	hsa-mir-145	flank5p(a-5)	flank	flank	flank	TN	TN	TN
chr5	159912417	159912418	C	G	18474871;18660546;<br>22711332	change in expr	hsa-mir-146a	mature3p(a+4)seed	seed	seed	seed	TP	TP	TP
chr5	168195355	168195356	G	A	23566829	change in expr	hsa-mir-218-2	flank5p(a-96)	arm	arm	arm	TP	TP	TP
chr6	52009266	52009267	C	T	18356149	no change	hsa-mir-206	flank3p(a+35)	flank	flank	flank	TN	TN	TN
chr7	129414552	129414553	A	G	22038834	change in expr	hsa-mir-96	mature3p(a+6)seed	seed	seed	seed	TP	TP	TP
chr7	129414573	129414574	A	G	22038834	no change	hsa-mir-96	loop(a+e1)	arm	arm	arm	FP	FP	FP
chr7	129414595	129414596	G	T	19363479	change in expr	hsa-mir-96	mature5p(a+6)seed	seed	seed	seed	TP	TP	TP
chr7	129414596	129414597	C	T	19363479;22038834	change in expr	hsa-mir-96	mature5p(a+5)seed	seed	seed	seed	TP	TP	TP
chr8	9760698	9760699	G	C	22430032	change in expr	hsa-mir-124-1	downstream(a+199)				FN	FN	FN
chr9	73424963	73424964	G	A	26056285	no change	hsa-mir-204	mature5p(a+5)seed	nochange	nochange	nochange	TN	TN	TN
chr9	96938219	96938220	C	A	16778182	no change	hsa-let-7a-1	flank5p(a-19)	flank	flank	flank	TN	TN	TN
chr9	139565149	139565150	A	G	20621067	change in expr	hsa-mir-126	flank3p(a+12)	arm	arm	flank	TP	TP	FN
chr10	53059405	53059406	T	C	25683625	change in expr	hsa-mir-605	arm3p(m+2)	nochange	nochange	nochange	FN	FN	FN
chr10	102734777	102734778	C	G	24743625	change in expr	hsa-mir-608	mature5p(a+22)	mature	mature	mature	TP	TP	TP
chr10	135061111	135061112	C	T	23334589	change in expr	hsa-mir-202	arm5p(m-15)	arm	arm	arm	TP	TP	TP
chr12	62997345	62997346	G	C	16778182	no change	hsa-let-7i	upstream(a-120)				TN	TN	TN
chr12	81329535	81329536	A	C	24503492	change in expr	hsa-mir-618	arm3p(l+25)	seed	seed	seed	TP	TP	TP
chr13	50623101	50623102	G	A	16251535	change in expr	hsa-mir-16-1	flank3p(a+7)	nochange	flank	nochange	FN	FN	FN
chr13	50623101	50623102	G	A	16251535	change in expr	hsa-mir-15a	downstream(a+153)				FN	FN	FN
chr16	69967004	69967005	C	A	20358594;22012839	change in expr	hsa-mir-140	arm5p(m-1e)	mature	mature	mature	TP	TP	TP
chr17	57918726	57918727	A	G	16778182	no change	hsa-mir-21	flank3p(a+29)	flank	flank	flank	TN	TN	TN
chr19	52196135	52196136	G	A	18356149	change in expr	hsa-let-7e	flank3p(a+19)	flank	flank	flank	FN	FN	FN
chr19	52196408	52196409	G	A	21788734	change in expr	hsa-mir-125a	flank5p(a-98)	flank	flank	flank	FN	FN	FN
chr19	52196452	52196453	T	C	21788734	no change	hsa-mir-125a	flank5p(a-54)	flank	flank	flank	TN	TN	TN
chr19	52196527	52196528	G	T	17400653	change in expr	hsa-mir-125a	mature5p(a+8)	seed	seed	seed	TP	TP	TP
chr21	17912066	17912067	C	G	16778182	no change	hsa-let-7c	flank5p(a-81)	flank	flank	flank	TN	TN	TN
chr22	46509539	46509540	C		16778182	no change	hsa-let-7b	flank5p(a-26)	flank	flank	flank	TN	TN	TN
chr22	46509676	46509677	G	C	16778182	no change	hsa-let-7b	flank3p(a+29)	mature	mature	mature	FP	FP	FP
chr22	46509701	46509702	G	A	16778182	no change	hsa-let-7b	flank3p(a+54)	nochange	nochange	nochange	TN	TN	TN
chrX	49779217	49779218	C	G	19568434;19617315	change in expr	hsa-mir-502	arm5p(m-3)	arm	arm	arm	TP	TP	TP
chrX	135633050	135633051	T	G	19617315	change in expr	hsa-mir-934	mature5p(a+e1)	mature	mature	mature	TP	TP	TP
chrX	145075803	145075804	C	G	19617315	change in expr	hsa-mir-890	arm3p(l+23)	arm	arm	arm	TP	TP	TP
chrX	145078732	145078733	A	G	19617315	change in expr	hsa-mir-892b	mature3p(a+15)	mature	mature	mature	TP	TP	TP
chrX	146353878	146353879	A	G	19568434;19617315	change in expr	hsa-mir-510	mature3p(a+2)seed	seed	seed	seed	TP	TP	TP
chrX	146353920	146353921	C	T	19617315	change in expr	hsa-mir-510	arm5p(m-4)	arm	arm	mature	TP	TP	TP
